# Role of the Lower and Upper Intestine in the Production and Absorption of Gut Microbiota-Derived PUFA Metabolites

**DOI:** 10.1371/journal.pone.0087560

**Published:** 2014-01-27

**Authors:** Céline Druart, Audrey M. Neyrinck, Bruno Vlaeminck, Veerle Fievez, Patrice D. Cani, Nathalie M. Delzenne

**Affiliations:** 1 Metabolism and Nutrition Research Group, Louvain Drug Research Institute, Université catholique de Louvain, Brussels, Belgium; 2 Laboratory for Animal Nutrition and Animal Product Quality, Faculty of Bioscience Engineering, Ghent University, Melle, Belgium; 3 WELBIO, Walloon Excellence in Life sciences and BIOtechnology, Université catholique de Louvain, Brussels, Belgium; Instutite of Agrochemistry and Food Technology, Spain

## Abstract

*In vitro* studies have suggested that isolated gut bacteria are able to metabolize PUFA into CLA (conjugated linoleic acids) and CLnA (conjugated linolenic acids). However, the bioavailability of fatty acid metabolites produced *in vivo* by the gut microbes remains to be studied. Therefore, we measured intestinal concentration and plasma accumulation of bacterial metabolites produced from dietary PUFA in mice, first injected with a lipoprotein lipase inhibitor, then force-fed with either sunflower oil (200 µl) rich in n-6 PUFA or linseed oil (200 µl) rich in n-3 PUFA. The greatest production of bacterial metabolites was observed in the caecum and colon, and at a much lesser extent in the jejunum and ileum. In the caecal content, CLA proportions were higher in sunflower oil force-fed mice whereas CLnA proportions were higher in linseed oil force-fed mice. The accumulation of the main metabolites (CLA *cis*-9,*trans*-11-18:2 and CLnA *cis*-9,*trans*-11,*cis*-15-18:3) in the caecal tissue was not associated with their increase in the plasma, therefore suggesting that, if endogenously produced CLA and CLnA have any biological role in host metabolism regulation, their effect would be confined at the intestinal level, where the microbiota is abundant.

## Introduction

The trillions of bacteria housed in our gastro-intestinal tract play an essential role in host homeostasis. This bacterial population was shown to be able to modulate the host's energy metabolism [1], inflammation [2], [3] and immunity [4]. The interactions between bacteria and their host are mediated, among others, by metabolites produced by the bacteria [5]. Due to the huge size of the microbiome, a very high metabolic potential of the gut microbiota could be envisaged. The contribution of bacteria to fatty acid metabolism has been mostly and largely studied in ruminants [6]. I*n vitro* studies showed that gut bacteria isolated from humans are also able to metabolize linoleic acid (LA) and α-linolenic acid (α-LnA) into metabolites like conjugated linoleic acids (CLA), conjugated linolenic acids (CLnA), and other trans fatty acids such as vaccenic acid (*trans*-11-18:1) [7]–[9]. These *in vitro* studies have shown that the main PUFA-derived bacterial metabolites are rumenic acid (CLA *cis*-9,*trans*-11-18:2), vaccenic acid (*trans*-11-18:1), CLnA *cis*-9,*trans*-11,*cis*-15-18:3 and *trans*-11,*cis*-15-18:2 [7], [8]. But these *in vitro* studies and the data from the biohydrogenation pathways of LA and α-LnA, which are quite well characterized in ruminants, suggest that other minor metabolites could also be produced such as CLA *trans*-10,*cis*-12-18:2; CLA *trans*-9,*trans*-11-18:2 and CLA *cis*-9,*cis*-11-18:2 [7], [9]–[11].

Interesting properties were attributed to dietary supplementation with chemically produced conjugated fatty acids isomers both in rodents and humans. Dietary supplementation with CLA (mainly *cis*-9,*trans*-11-18:2 or *trans*-10,*cis*-12-18:2 or a combination of both) was associated with anti-obesity, anti-atherogenic and anti-inflammatory properties [12], [13]. CLA also inhibited growth of cancer cells *in vitro* [14]. Similarly, CLnA, produced by *Bifidobacterium breve*, were able to inhibit the growth of human colon cancer cells and could be associated with an anti-obesity effect [15], [16]. However, some effects of both CLA and CLnA seem to be isomer-specific and the conclusion about these effects should be cautiously interpreted [17]–[20]. In addition to these health effects attributed to CLA and CLnA, it seems that other bacterial metabolites such as vaccenic acid and very long chain conjugated PUFA could also be associated with beneficial effects towards the host [21], [22].

In fact, CLA-producing bacteria are detected in the human gut microbiota, suggesting that PUFA-derived bacterial metabolites production may take place in the human gut. *In vitro* studies confirm this, as bacteria isolated from human gut are able to produce both CLA and CLnA [7]–[9]. Our hypothesis is that metabolites endogenously produced by the gut microbiota from dietary PUFA could be a new kind of metabolites able to influence host's physiology. We proposed to test this hypothesis in mice, which possess in their gut microbiota, the bacteria able to produce PUFA metabolites in both human and animals (Lactobacilli, Bifidobacteria, Roseburia) [9].

In a previous study, we have shown that CLA can be found in the liver and adipose tissue of mice fed a high-fat (HF) diet, and that the dietary supplementation of fermentable carbohydrates with prebiotic properties, modulates proportion of CLA and lipid derived metabolites in the adipose tissue [23]. This suggests that CLA can be endogenously produced *in vivo*, but there is no clear view of the availability of the PUFA-derived bacterial metabolites, and of the nature of the fatty acid metabolites that can be produced from different fatty acid precursors (n-3 versus n-6 PUFA, i.e.).

One challenging question raised by our previous results is to identify whether bacterial metabolites are produced in the upper and/or in the distal part of the gut, and if they can be absorbed. The upper parts of the gut contain fewer bacteria than the distal parts of the gut (10^3^–10^4^ bacteria/gram of jejunum content versus 10^11^–10^12^ bacteria/gram of colon content) [24]–[26]. However, the jejunum remains the main site of fatty acid absorption [27]. The distal parts of the gut contain the highest number of bacteria but the potential contribution of the caecum and colon to fatty acid absorption remains poorly studied.

The aim of this study was to investigate the production of PUFA-derived bacterial metabolites in the contents and tissues of different sites of the intestine of mice having received a single oral load of either linseed oil (rich in n-3 PUFA) or sunflower oil (rich in n-6 PUFA). PUFA-derived bacterial metabolites absorption and bioavailability has been assessed by analyzing fatty acid metabolites accumulation in the blood after pharmacological blocking of lipoprotein lipase activity (Tyloxapol) to avoid tissue uptake and distribution.

## Materials and Methods

### Ethics Statement

The ethical committee for animal care of the Health Sector of the Université catholique de Louvain, under the supervision of prof. F. Lemaigre et J.P. Dehoux under the specific number 2010/UCL/MD022, has specifically approved the animal experiments performed in this study. Housing conditions were as specified by the Belgian Law of 29 May 2013, on the protection of laboratory animals (agreement n° LA 1230314).

### Animals and diets

Male C57bl6/J mice (Charles River Laboratories, France) of 9 weeks old at the beginning of the experiment, were housed in a controlled environment (12 h daylight, lights off at 6p.m.) with free access to diet (AO4, SAFE, Villemoisson-sur-Orge, France) and water. After one week of acclimatization and a 16-h period of fasting, mice were injected with the lipoprotein lipase (LPL) inhibitor Tyloxapol (iv retro-orbital injection, 0.5 mg/g of body weight; Sigma-Aldrich) under light anesthesia (Isoflurane; Forene, Abbott, Queenborough, Kent, England). Tyloxapol injection results in an accumulation of lipids in the blood [28]. This accumulation of lipids should allow to assess the fatty acid profile of the circulating lipids in relation to their absorption rate. Thirty minutes after Tyloxapol injection, mice were force-fed with vegetable oils (200 µl). Six mice received sunflower oil and the other six received linseed oil. We analyzed the fatty acid profile of both vegetable oils by gas chromatography equipped with a flame-ionization detector (GC-FID) (Table 1).

**Table 1:** Fatty acid profile of linseed and sunflower oils.

	Linseed Oil	Sunflower Oil
16:0	5,42	6,53
*cis*-9-16:1	0,11	0,14
18:0	4,10	3,15
*cis*-9-18:1	22,04	24,38
*cis*-11-18:1	0,70	0,77
*cis*-9,*trans*-12-18:2	ND	0,39
*trans*-9,*cis*-12-18:2	ND	0,33
*cis*-9,*cis*-12-18:2 (LA)	15,80	63,12
20:0	0,22	0,27
*cis*-9,*cis*-12,*cis*-15-18:3 (α-LnA)	50,79	0,06
*trans*-9,*trans*-11-18:2	ND	0,04
*cis*-9-20:1	0,25	0,21
22:0	0,18	0,68

The results are expressed as a percentage of the identified fatty acids (n.d.: not detectable, below the level of detection). Only the isomers above the limit of detection were presented in this Table 1.

### Blood and tissue samples

Blood from the tail vein was sampled before the Tyloxapol injection (T-30), before oil force-feeding (T0), and ten minutes (T10) and 2 hours (T120) after oil force-feeding. Four hours after force-feeding (T240), mice were anesthetized with isoflurane gaz (Forene, Abbott, Queenborough, Kent, England). Blood from portal vein and cava vein was harvested for further analysis. The blood was centrifuged (5 min, 13000 g) and plasma was stored at -20°C. Mice were killed by cervical dislocation. Jejunum was sampled 4 cm after the stomach, ileum was sampled just before the caecum and the colon was sampled just after the caecum. Intestinal contents were carefully collected and frozen in liquid nitrogen before storage at −80°C. Then, all intestinal tissue samples (jejunum, ileum, caecum and colon) were flushed and carefully washed with cold saline solution (NaCl 0.9%) before being frozen in liquid nitrogen and storage at −80°C.

### Fatty acid profile analysis

To determine the fatty acid profile in intestinal tissues and intestinal contents, we weighed 25 mg of jejunum, ileum and colon contents, 50 mg of caecal content and 50 mg of intestinal tissues (jejunum, ileum, caecum and colon). To determine the fatty acid profile in blood, 50 µl of plasma was used. The tissues, contents and blood samples were homogenized in a methanol:chloroform mixture (1∶2 V/V). Homogenates were filtered with Whatman filters N°1 (porosity 10 µm). The filters were rinsed with 2 ml of chloroform and 1 ml of methanol. Homogenates were purified successively with KCl 0.88% and KCl 0.88%:methanol (1∶1 V/V). After centrifugation (1500 g, 5 min), the chloroform phase was collected in new tubes and evaporated under a nitrogen flux.

The esterified fatty acids were then subjected to an alkaline hydrolysis (saponification). For that purpose, a solution of KOH in methanol was added and incubated at 70°C for 1 hour. The free fatty acids were methylated as follows: 0.4 ml of HCl in methanol (1.2 M) was added in the tube and incubated at 70°C for 20 minutes. Fatty acid methyl esters (FAME) were then extracted with hexane.

To determine the fatty acid profile in oils, 15 mg of oil was subjected to an alkaline hydrolysis and the free fatty acids were methylated following the procedures described above.

Quantification of FAME was made by gas-liquid chromatography (Focus GC, Thermo-Finnigan, Interscience, Belgium). The chromatograph was equipped with a flame ionization detector and a 100 m capillary column (i.d. 0.25 mm, film thickness 0.20 µm; RT-2560, Restek, Interscience, Belgium) using H_2_ as the carrier gas at a constant flow of 1.5 ml/min. The initial oven temperature was 80°C, increased at 25°C/min to 175°C (held for 10 min), then increased at 1°C/min to 200°C (held for 15 min), then increased at 5°C/min to 215°C (held for 5 min) and finally decreased at 20°C/min to 80°C. The temperature of the flame ionization detector was maintained at 250°C. The identification of each peak was made by comparison of retention times with pure FAME standards (Larodan Fine Chemicals AB, Malmö, Sweden). Peak identification was further confirmed by an analysis of samples (oils, caecum contents and caecum tissues) using a GC method combining two different temperature programs following the procedure described below. Composition analysis of FAME was carried out by a gas chromatograph (HP 6890A, Agilent Technologies, Diegem, Belgium) equipped with a 75-m SP-2560™ capillary column (i.d. 0.18 mm, film thickness, 0.14 µm; Supelco Analytical, Bellefonte, PA) and a flame ionization detector. A combination of two oven temperature programs was used in this study to achieve determination of most *cis* and *trans* 16∶1, branched chain fatty acids and 18∶1 isomers according to the method of Kramer et al. [29] with modifications [30]. Most FAME peaks were identified using quantitative mixtures of methyl ester standards (BR2 and BR3, Larodan Fine Chemicals, Malmö, Sweden; Supelco® 37, Supelco Analytical, Bellefonte, PA; PUFA-3, Matreya LLC, Pleasant Gap, PA). Fatty acids for which no standards were available commercially were identified by order of elution according to Precht et al. [31] and Kramer et al. [29]. Also, aliquots of methylated samples were pooled and subsequently fractionated by TLC on silica gel plates impregnated with silver nitrate according to Destaillats et al [32] with modifications, in order to confirm purity for peaks of interest in chromatograms and to eliminate peaks that were not fatty acids (e.g. dimethyl acetals).

### Blood lipids analysis

Plasma triglycerides and non-esterified fatty acids (NEFA) concentrations were measured using kits coupling enzymatic reaction and spectrophotometric detection of reaction end-products (Diasys Diagnostic and Systems, Germany and Randox Laboratories Limited, United Kingdom), according to the manufacturer's instructions.

### Statistical analysis

Results are presented as mean ± SEM (standard error of mean). Linear mixed-effects model followed by contrasts assessed the statistical difference between both the two different oil force-feedings and the different intestinal contents or the different intestinal tissues or the different blood sample times (SPSS, version 20.0). For the fatty acid analyzed only in linseed oil force-fed mice, the statistical significance of difference between the different intestinal contents, intestinal tissues or blood sample times were assessed by one-way ANOVA followed by Bonferroni's post-hoc multiple comparison test (GraphPad Prism Software). Statistical significance was defined for a p-value lower than 0.05.

## Results

### Fatty acid characterization of linseed and sunflower oils

Sunflower oil was selected upon its high content in n-6 PUFA (LA) and the linseed oil for its high content in n-3 PUFA (α-LnA) [33]. GC-FID analysis of the oils confirmed that α-LnA represents about 50% of the fatty acids identified in the linseed oil whereas it represents only 0.06% of the fatty acids identified in the sunflower oil (Table 1). LA represents 63% of the total fatty acids identified in the sunflower oil whereas it represents only 16% of the total fatty acids identified in the linseed oil (Table 1). The linseed oil is thus characterized by a low ratio LA/α-LnA (0.3) whereas the sunflower oil is characterized by a high ratio LA/α-LnA (1052). Sunflower oil contained a higher proportion of *trans*-9,*cis*-12-18:2, which represented 0.3% of the total fatty acids, as well as a higher proportion of CLA *trans*-9,*trans*-11-18:2 than the linseed oil. These observations will be taken into account when interpreting results obtained in tissues and biological fluids.

### Fatty acid profile in intestinal contents of oil force-fed mice reveals a high availability of PUFA precursors throughout the gut and a higher proportion of bacterial metabolites in the caecum and colon than in the jejunum or ileum

In the intestinal contents, the total fatty acid amount decreased with progress down the gut. The total amount of fatty acid was very high in the jejunum (117.9 µg fatty acids/mg of jejunum content in linseed oil force-fed mice and 90.69 µg fatty acids/mg of jejunum content in sunflower oil force-fed mice) and decreased dramatically between jejunum and ileum (38 µg fatty acids/mg of ileum content in linseed oil force-fed mice and 39.74 µg fatty acids/mg of ileum content in sunflower oil force-fed mice). The amounts of fatty acid found in ileum, caecum and colon contents were similar.

**Figure 1:**
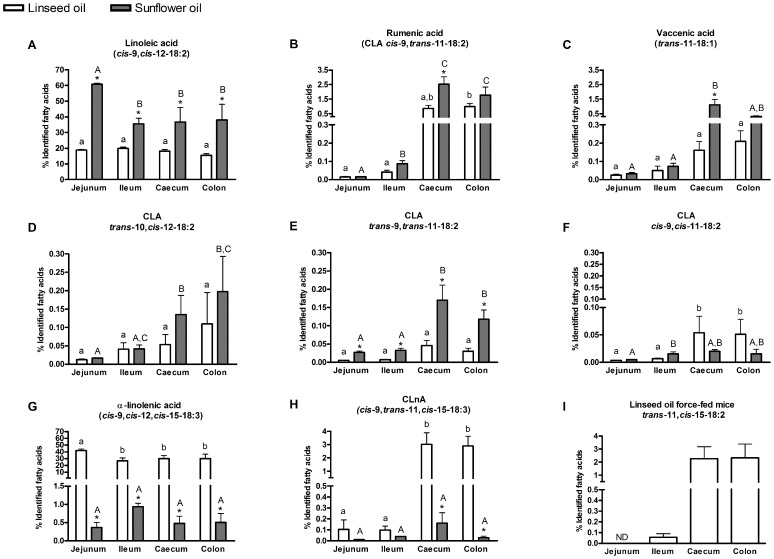
Fatty acid profile in intestinal contents. Linoleic acid, α-linolenic acid and PUFA-derived bacterial metabolites in intestinal contents (jejunum content – ileum content – caecum content and colon content) of mice force-fed with linseed oil (rich in n-3 LnA) or sunflower oil (rich in n-6 LA). Results are expressed as a percentage of identified fatty acids. Data are mean ± SEM. Linear mixed-effects model followed by contrasts assessed the statistical difference between both the two oil force-feedings and the different intestinal contents. * superscripts show significant differences (p<0.05) between linseed oil force-feeding and sunflower oil force-feeding in each intestinal content. Different small superscript letters show significant differences (p<0.05) between intestinal contents in linseed oil force-fed mice. Different capital superscript letters indicate significant differences (p<0.05) between intestinal contents in sunflower oil force-fed mice. *trans*-11,*cis*-15-18:2 was detected in linseed oil force-fed mice only.

In all the different intestinal contents (jejunum, ileum, caecum and colon), varying amounts of LA and α-LnA were observed, in accordance with the fatty acid profile of the ingested oil: a high proportion of LA was observed in the group of mice that received sunflower oil, and a high proportion of α-LnA in the group of mice receiving linseed oil (Fig 1 A, G). Interestingly, oils force-feeding increased the contents of LA and α-LnA at a similar extent in the upper (jejunum and ileum contents) and lower (caecum and colon contents) parts of the gut (Fig 1 A, G), suggesting that the quantity of PUFA available for the bacterial metabolism was increased in all intestinal contents after administration of oils.

Interestingly, LA and α-LnA-derived bacterial metabolites are present in higher proportions in the content of the distal parts of the gut (caecum and colon) than in the content of the proximal parts of the gut (jejunum and ileum) (Fig 1 B-F, H-I).

The proportions of LA metabolites (such as rumenic acid, vaccenic acid, CLA *trans*-10,*cis*-12-18:2 and CLA *trans*-9,*trans*-11-18:2) were higher in the group of mice force-fed with sunflower oil compared with the mice force-fed with linseed oil (Fig 1 B-E). The proportions of α-LnA metabolites (such as CLnA *cis*-9,*trans*-11,*cis*-15-18:3 and *trans*-11,*cis*-15-18:2) were higher in the group of mice force-fed with linseed oil than in the group of mice force-fed with sunflower oil (Fig 1 H,I; Figure S1). However, the GC method with a single temperature program did not allow to clearly separate the *trans*-11,*cis*-15-18:2 and the *trans*-9,*cis*-12-18:2. With the two temperatures method, we observed that *trans*-9,*cis*-12-18:2 was not detectable in caecum content and caecum tissue of linseed oil force-fed mice whereas it was detectable in the sunflower oil force-fed mice, probably because it was supplied directly by the sunflower oil (Figure S1 and Figure S2). As *trans*-9,*cis*-12-18:2 was not detectable in caecum content and caecum tissue of the linseed oil force-fed mice, we assumed that *trans*-11,*cis*-15-18:2 was correctly identified only in this group of mice.

The monounsaturated bacterial metabolite called vaccenic acid (*trans*-11-18:1) can be produced from both LA and α-LnA. In the group of mice force-fed with sunflower oil, the proportion of vaccenic acid in the caecal content was higher than in the three other intestinal contents (jejunum, ileum and colon contents) (Fig 1 C). The vaccenic acid proportion in the caecal content was higher in the sunflower oil force-fed mice than in the linseed oil force-fed mice (Fig 1 C). This result could suggest that vaccenic acid is mainly produced from bacterial metabolism of LA rather than from bacterial metabolism of α-LnA. In other intestinal contents, we did not observe any difference of vaccenic acid content between the two groups of mice (Fig 1 C).

### Fatty acid profile in intestinal tissues

**Figure 2:**
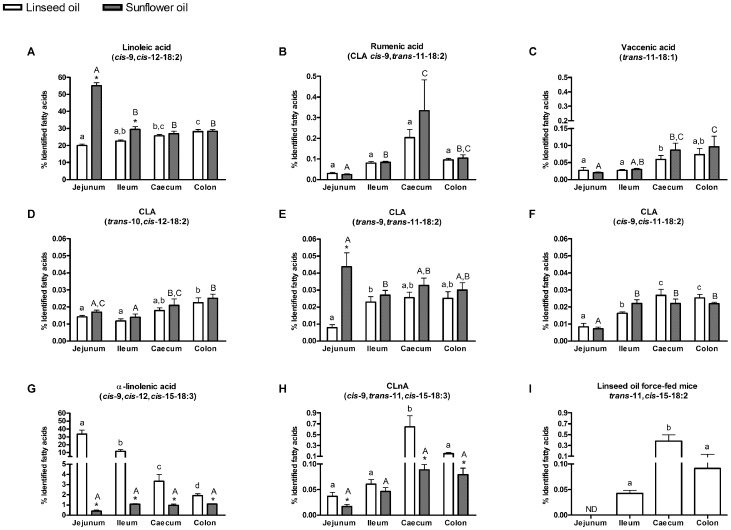
Fatty acid profile in intestinal tissues. Linoleic acid, α-linolenic acid and PUFA-derived bacterial metabolites in intestinal tissues (jejunum tissue – ileum tissue – caecum tissue and colon tissue) of mice force-fed with linseed oil (rich in n-3 LnA) or sunflower oil (rich in n-6 LA). Results are expressed as a percentage of identified fatty acids. Data are mean ± SEM. Linear mixed-effects model followed by contrasts assessed the statistical difference between both the two oil force-feedings and the different intestinal tissues. * superscripts show significant differences (p<0.05) between linseed oil force-feeding and sunflower oil force-feeding in each intestinal tissue. Different small superscript letters show significant differences (p<0.05) between intestinal tissues in linseed oil force-fed mice. Different capital superscript letters indicate significant differences (p<0.05) between intestinal tissues in sunflower oil force-fed mice. *trans*-11,*cis*-15-18:2 was detected in linseed oil force-fed mice only.

As shown in intestinal contents, and in accordance with the oil composition, the proportion of LA was higher in the sunflower oil force-fed mice than in the linseed oil force-fed mice, but this difference was significant in the jejunum and ileum tissues only (not in the caecum and colon tissues) (Fig 2 A). The proportion of α-LnA was higher in the linseed oil force-fed mice than in the sunflower oil force-fed mice in all intestinal tissues (Fig 2 G).

The different bacterial metabolites reached the intestinal tissues. Most of metabolites accumulated in the lower gut (from ileum to the colon) (Fig 2 B-D, F, H, I). Rumenic acid (CLA *cis*-9,*trans*-11-18:2), and vaccenic acid (*trans*-11-18:1), two main LA-derived bacterial metabolites, and CLnA *cis*-9,*trans*-11,*cis*-15-18:3 and *trans*-11,*cis*-15-18:2, two main α-LnA-derived bacterial metabolites, accumulated mainly in the caecal tissue (Fig 2 B,C; H,I). The proportions of *trans*-11,*cis*-15-18:2 and of CLnA *cis*-9,*trans*-11,*cis* -15-18:3 (to a lesser extent) mostly increased in the caecal tissue of linseed oil force-fed mice, and not in the colon (Fig 2 H,I) even if the proportion in the caecum and colon contents were similar. For none of the intestinal tissues, proportions of LA metabolites (rumenic acid and vaccenic acid) differed between the two groups of mice (Fig 2 B-D,F). The proportion of the α-LnA metabolite (CLnA *cis*-9,*trans*-11,*cis*-15-18:3) measured in the jejunum, caecum and colon tissues was higher in the linseed oil compared with the sunflower oil treated mice (Fig 2 H). Trans fatty acid isomers found in the original oil (e.g. *trans*-9,*trans*-11-18:2 and *trans*-9,*cis*-12-18:2 in sunflower oil) mainly accumulated in the jejunum tissue of sunflower oil force-fed mice (Fig 2E; Figure S1B), suggesting that these dietary fatty acids are mainly absorbed in the jejunum.

### Circulating Fatty acid profile

**Figure 3:**
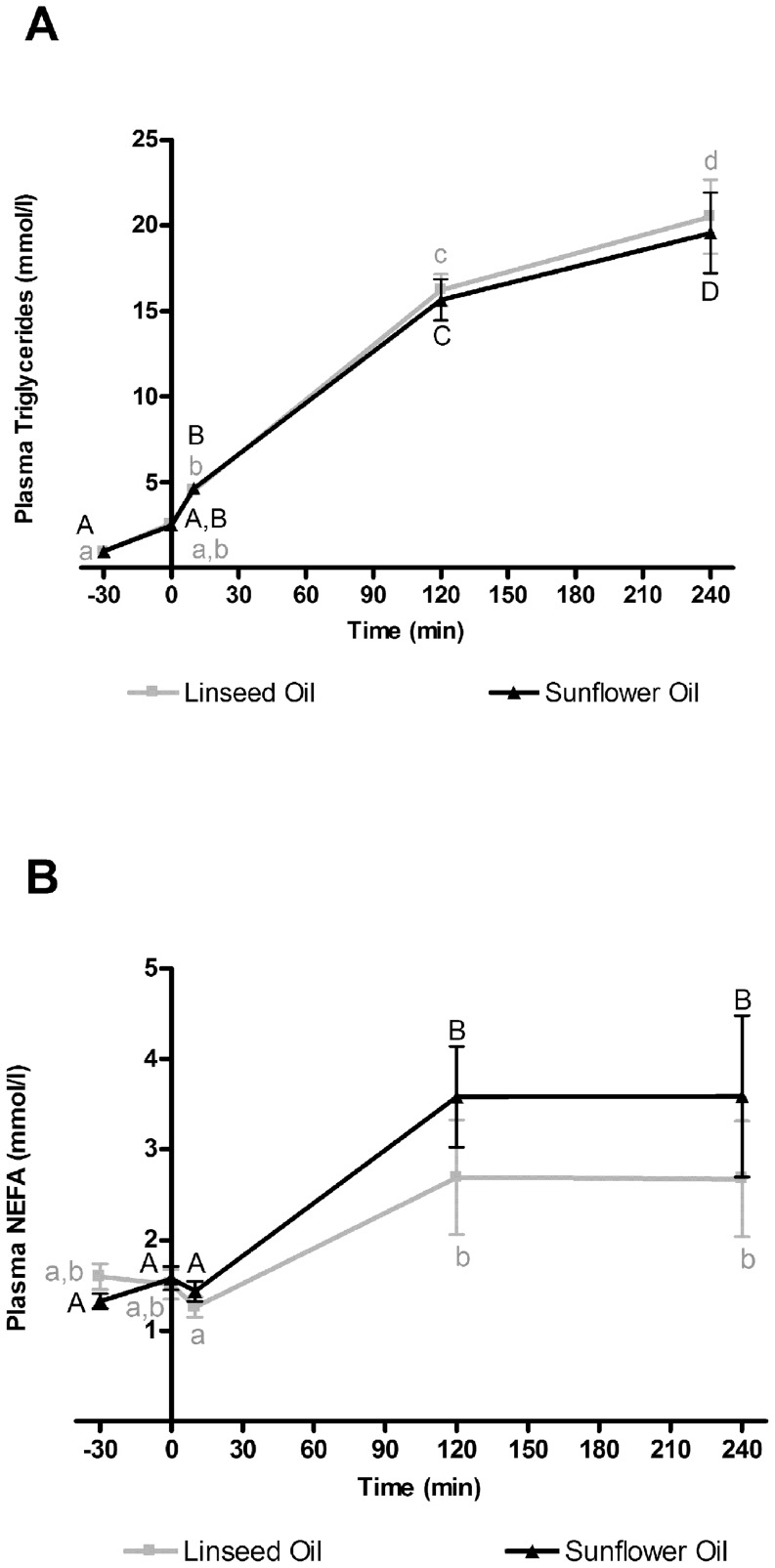
Blood lipids accumulation. Triglycerides and non-esterified fatty acids (NEFA) were measured just before the Tyloxapol injection (T-30), just before oil force-feeding (T0), ten minutes (T10), 2 hours (T120) and 4 hours (T240) after oil force-feeding in the plasma of mice force-fed with linseed oil (rich in n-3 LnA) or sunflower oil (rich in n-6 LA). Data are mean ± SEM. Linear mixed-effects model followed by contrasts assessed the statistical difference between both the two oil force-feedings and the blood sample times. Differences between linseed oil force-fed mice and sunflower oil force-fed mice were not significant at any of the blood sample times. Grey small superscript letters show significant differences (p<0.05) between blood sample times in linseed oil force-fed mice. Black major superscript letters show significant differences (p<0.05) between the blood sample times in sunflower oil force-fed mice.

In order to know if the bacterial metabolites found in the intestinal contents and in the intestinal tissues can exert some systemic effects, we analyzed the availability of these lipophilic metabolites in the blood. Mice were previously treated with LPL inhibitor, Tyloxapol, allowing lipid accumulation in the blood. The accumulation of lipids in blood is shown in Fig 3A (plasma triglycerides) and 3B (plasma NEFA). Plasma triglycerides increased directly after the Tyloxapol injection and until 240 min whereas the plasma NEFA increased immediately after the oil force-feeding and did not further increased between 120 min and 240 min (Fig 3 A, B).

**Figure 4:**
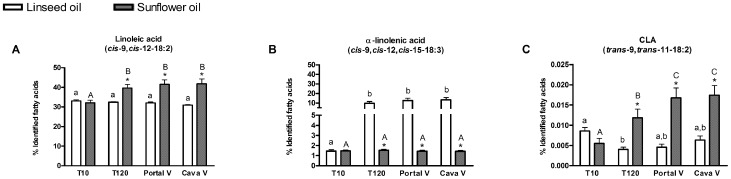
Fatty acid profile in circulating lipids. Linoleic acid, α-linolenic acid and PUFA-derived bacterial metabolites in peripheral plasma of mice force-fed with linseed oil (rich in n-3 LnA) or sunflower oil (rich in n-6 LA) at different timing after the force-feeding (10 minutes; 120 minutes and 240 minutes after the force-feeding). Results are expressed as a percentage of identified fatty acids. Data are mean ± SEM. Linear mixed-effects model followed by contrasts assessed the statistical difference between both the two oil force-feedings and the blood sample times. * superscripts show significant differences (p<0.05) between linseed oil force-feeding and sunflower oil force-feeding at each blood sample time. Different small superscript letters show significant differences (p<0.05) between blood sample times in linseed oil force-fed mice. Different capital superscript letters indicate significant differences (p<0.05) between blood sample times in sunflower oil force-fed mice. *trans*-11,*cis*-15-18:2 was detected in linseed oil force-fed mice only.

Ten minutes after force-feeding, plasma levels of LA and α-LnA were similar in both groups of mice. Later, i.e. 120 and 240 minutes after force-feeding, the fatty acid profile in the circulating lipids was in agreement with the fatty acid profile of the oils: we found a higher proportion of LA in the blood of sunflower oil force-fed mice and a higher proportion of α-LnA in the blood of linseed oil force-fed mice (Fig 4 A, B).

Proportions of the three bacterial metabolites that accumulated mainly in the caecal tissue (CLA *cis*-9,*trans*-11-18:2, CLnA *cis*-9,*trans*-11,*cis*-15-18:3 and *trans*-11,*cis*-15-18:2) were not modified by oil force-feeding, nor by the timing of the different blood samplings (Data not shown). However, the proportions of CLA *trans*-9,*trans*-11-18:2 and *trans*-9,*cis*-12-18:2, fatty acids that accumulated in the jejunum tissue and were probably of dietary origin, increased in a time-dependent way (Fig 4C, Fig S1C).

## Discussion

We have previously shown that proportions of rumenic acid and vaccenic acid were increased in caecal tissue and subcutaneous adipose tissue of mice fed a HF diet or a HF diet supplemented with prebiotic carbohydrates, that modulate the gut microbiota composition [23], [34]. This study suggests that those particular fatty acids may be metabolites produced from dietary fatty acids by commensal bacteria and that those metabolites could have an effect outside the gut. However, little is known about the availability of dietary fatty acids in the distal parts of the gut and the systemic bioavailability of PUFA-derived bacterial metabolites. In the present *in vivo* experiment, we analyzed the fatty acid profile in all the intestinal contents and intestinal tissues, in order to verify if the precursors, LA and α-LnA, were effectively present in all the intestinal contents, including the distal parts (caecum and colon contents), where bacteria are the most abundant. A high proportion of LA and α-LnA was observed in mice receiving sunflower oil (rich in n-6 LA) and linseed oil (rich in n-3 LnA), respectively; these changes occurring in all intestinal contents (including caecum and colon contents). Although it is assumed that 95% of dietary lipids are absorbed in the proximal part of the gut in physiologic conditions [27], our results suggest that oils were not completely absorbed in the jejunum and ileum under our experimental conditions. In a HF diet model, it has been shown that an overflow of lipids reaches the distal part of the small intestine, and so could also reach the colon [35]. Therefore, the oil force-feeding is not representative for physiologic conditions but rather mimics an overflow of lipids, as created in mice fed a HF diet. Our protocol created a sufficient flow of substrates available for bacterial metabolism in all intestinal segments.

Since all mice arrived at the same time, had the same genetic background, ate the same food from weaning, and were randomly assigned to one or the other oil before the force-feeding experiment, we suppose that microbiota composition did not differ significantly between the two groups of mice before the experiment. Indeed, major differences in the composition of the gut microbiota composition could have important impact on PUFA-derived bacterial metabolites production. However, modifications of the gut microbiota composition could be induced by the oil force-feedings. Indeed, arguments from the literature suggest that the type of dietary fat is able to modulate the composition of the gut microbiota [35]–[37]. It was shown that saturated fatty acids induced more changes in the gut microbiota composition than monounsaturated fatty acids or polyunsaturated fatty acids [35]. Mujico *et al* have also shown that supplementation with an oleic acid-derived compound is able to decrease body weight gain and to restore the proportions of bacteria after HF diet treatment [37]. However, in this short-term experiment, we did not know if 4 hours are sufficient to change the gut microbiota composition. So, the analysis of the gut microbiota composition after the oil force-feeding represents a very interesting prospect to the present study.

This study highlights for the first time that a production of CLnA takes place *in vivo* and in agreement with the *in vitro* studies, the main α-LnA metabolites seem to be CLnA *cis*-9,*trans*-11,*cis*-15-18:3 and *trans*-11,*cis*-15-18:2 [8], [15]. Proportions of the majority of bacterial metabolites (LA metabolites and α-LnA metabolites) were lower in the contents of the proximal parts of the gut (jejunum and ileum contents) than in the contents of the distal parts of the gut (caecum and colon contents) where the density and the diversity of the gut microbiota are the highest [24], [38], [39]. In view of these results, we postulate that these metabolites are produced by bacterial metabolism rather than supplied by the diet.

In the caecum content, the production of PUFA-derived bacterial metabolites is influenced both by the quantity and the nature (n-3 PUFA versus n-6 PUFA) of the dietary fatty acids. Indeed, we found higher proportions of CLnA *cis*-9,*trans*-11,*cis*-15-18:3 and *trans*-11,*cis*-15-18:2 in the mice force-fed with linseed oil (rich in n-3 PUFA) whereas we observed a higher proportion of rumenic acid (CLA *cis*-9,*trans*-11-18:2), vaccenic acid (*trans*-11-18:1) and CLA *trans*-9,*trans*-11-18:2 in mice force-fed with sunflower oil (rich in n-6 PUFA). These observations were already found in ruminants where the biohydrogenation pathways of LA and α-LnA are quite well characterized [40], suggesting that a similar “biohydrogenation pathway” could take place in mammalian gut, even if the two microbial environments are different.

The production of one particular PUFA-derived bacterial metabolite, CLA *cis*-9,*cis*-11-18:2, seems to be influenced by both the intestinal segment and the nature of the dietary fatty acid. Indeed, the proportion of CLA *cis*-9,*cis*-11-18:2 in the ileum content was higher in the sunflower oil force-fed mice than in the linseed oil force-fed mice whereas its proportion in the contents of the distal parts of the gut (caecum and colon contents) tended to be higher in the linseed oil force-fed mice than in the sunflower oil force-fed mice. According to the ruminant biohydrogenation pathways proposed by Chilliard Y. et *al*, the CLA *cis*-9,*cis*-11-18:2 could be a metabolite produced from both LA and α-LnA [6]. This observation could explain the differences between the intestinal segments since the bacterial population of the different intestinal segments seems to be different [41].

Our results suggest that PUFA-derived bacterial metabolites are mainly produced in the distal part of the gut. However, the main place of lipid absorption remains the small intestine [27]. We confirm this since a two-fold decrease of total fatty acid amount was observed between jejunum and ileum contents; suggesting that a large part of the fatty acids present in the intestinal lumen are absorbed in the jejunum. To study the bioavailability of PUFA-derived bacterial metabolites, we focused on their accumulation in intestinal tissues and in peripheral plasma. In the intestinal tissues, the majority of the bacterial metabolites accumulated in the caecum tissue which is in agreement with the higher proportion of these metabolites in the caecum content. However, the proportions of some bacterial metabolites such as rumenic acid (CLA *cis*-9,*trans*-11-18:2), CLnA *cis*-9,*trans*-11,*cis*-15-18:3 and *trans*-11,*cis*-15-18:2 were similar in the caecum and colon contents. In intestinal tissues, the proportions of these bacterial metabolites were higher in the caecum tissue than in the colon tissue, suggesting that the caecal tissue has a higher capacity of up-take than the colon tissue. This observation could be explained by the thicker layer of mucus in the colon than in the caecum [42]. The mucus layer could interfere with the fatty acid up-take by intestinal cells. However, for these bacterial metabolites that accumulated mainly in the caecum tissue, their bioavailability, revealed by analysis of circulating lipids, was not influenced neither by the type of oil used nor by the timing of blood samples. These results suggest that these bacterial metabolites could exert rather local effects in intestinal tissues of host than direct systemic effects.

However, changes in fatty acid profile in intestinal tissues could have effects on host's physiology, namely by interactions with cell membrane receptors like G protein-coupled receptors (GPR) or with nuclear receptor such as peroxisome proliferator-activated receptors (PPAR). On one hand, it was shown that several GPRs, such as GPR40 and GPR120, which are expressed in the gastrointestinal tissues, could be considered as fat sensors [43]. Schmidt *et al* have shown that two CLA isomers, CLA *cis*-9,*trans*-11-18:2 and CLA *trans*-10,*cis*-12-18:2 are full GRP40 agonists [44]. Activation of GPR40 in enteroendocrine cells increases incretins production by these cells [45]. GPR120 was also shown to be able to increase incretins production after activation by lipid agonist, such as linoleic and α-linolenic acid [46]. However, the ability of CLA or CLnA to bind GPR120 has not been demonstrated yet. It could be very interesting to test the hypothesis that PUFA-derived bacterial metabolites, by acting as GPR40 and/or GPR120 agonists, could thus modulate host metabolism, namely by changing incretins production. On the other hand, PPARγ is expressed at the intestinal level and activation of this receptor by CLA is associated with a decreased of inflammation in murine model of inflammatory bowel diseases [47]. So, the activation of these receptors by PUFA-derived bacterial metabolism at the intestinal level could have an effect on host metabolism or host inflammation.

In a previous study, we described an accumulation of PUFA-derived bacterial metabolites in adipose tissue [34]. It might be possible that under pathologic conditions characterized by an alteration of the gut barrier function such as in HF diet conditions [48] or inflammatory bowel diseases [24], these PUFA-derived bacterial metabolites could cross the gut epithelium and reach the systemic bloodstream in a higher quantity than under physiologic conditions. Indeed, it has been shown that HF diet treatment increases gut permeability and thereby promotes the paracellular translocation of lipophilic molecules, such as lipopolysaccharides (LPS), lipophilic components of the gram-negative bacterial cells [48]. Under HF diet treatment, it is likely that other lipophilic bacterial metabolites (e.g. CLA and CLnA) could cross the gut epithelium in this way and reach the lymphatic or portal circulation.

Contrary to the PUFA-derived bacterial metabolites that were undetectable in oils and that accumulated mainly in the caecum tissue, two fatty acids, CLA *trans*-9,*trans*-11-18:2 and *trans*-9,*cis*-12-18:2, accumulated mainly in the jejunum tissue of sunflower oil force-fed mice probably because these fatty acids are supplied by the sunflower oil. This suggests that the jejunum tissue fatty acid profile reflects the oil fatty acid profile rather than an accumulation of PUFA-derived bacterial metabolites produced *in situ*. In circulating lipids (120 and 240 minutes after force-feeding), the proportions of both fatty acids are higher in the sunflower oil force-fed mice than in the linseed oil force-fed mice. Furthermore, in the sunflower oil force-fed mice, the proportion of these fatty acids increased in a time dependent manner, suggesting that they are absorbed from the jejunum. We propose that among the fatty acids present in the diet, or produced upon bacterial metabolism, only those accumulating in the jejunum tissue can be effectively absorbed. Thus our data suggests that the systemic bioavailability of the PUFA-derived bacterial metabolites produced in the lower part of the gut remains negligible.

**Figure 5:**
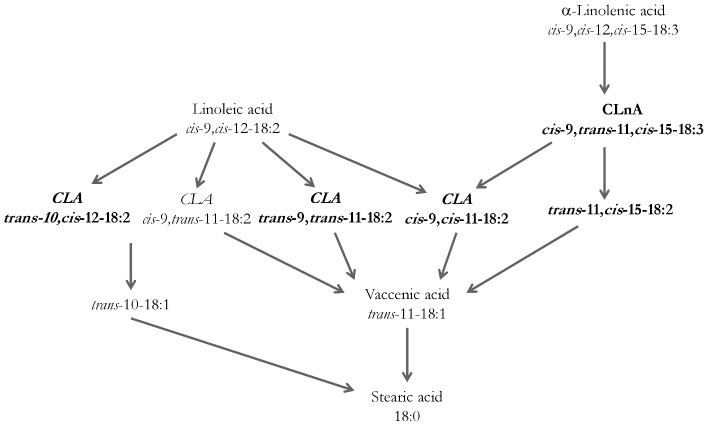
Proposed metabolic pathway of linoleic acid and α-linolenic acid by murine gut microbiota. PUFA-derived bacterial metabolites in bold characters were not described in the previous study [23].

Finally, in view of the results obtained in this experiment, we are able to validate and extend our knowledge on gut microbial metabolism occurring *in vivo* in rodents from dietary PUFA. Before our studies, the proposed pathways were suggested from *in vitro* studies, or extrapolated from studies performed in ruminants [6], [7], [11], [23] (Fig 5). The relevance of those pathways in humans would be interesting to evaluate in the future.

## Supporting Information

Figure S1 **Sum of** ***trans*** **-9,** ***cis*** **-12-18:2 and** ***trans*** **-11,** ***cis*** **-15-18:2 in intestinal contents, intestinal tissues and circulating lipids of the sunflower oil force-fed mice.** The results are expressed as a percentage of identified fatty acids. Data are mean ± SEM. Statistical significance of differences between intestinal contents, intestinal tissues and blood samples times were assessed by one-way ANOVA followed by Bonferroni's post-hoc multiple comparison test. Values with unlike superscript letters are significantly different (p<0.05).
(TIF)

Figure S2 ***trans*** **-9,** ***cis*** **-12-18:2 and** ***trans*** **-11,** ***cis*** **-15-18:2 in caecum content and caecum tissue analyzed by the GC method combining two temperature program.** The results are expressed as a percentage of identified fatty acids. Data are mean ± SEM. Statistical significance of difference between oil force-feedings was assessed by Student t-test (** p<0.01; *** p<0.001).
(TIF)
