# Supplementary figures and images for: Role of the Lower and Upper Intestine in the Production and Absorption of Gut Microbiota-Derived PUFA Metabolites
Source: PLoS One. 2014 Jan 27;9(1):e87560. doi: 10.1371/journal.pone.0087560 (PMC3903770; doi:10.1371/journal.pone.0087560)

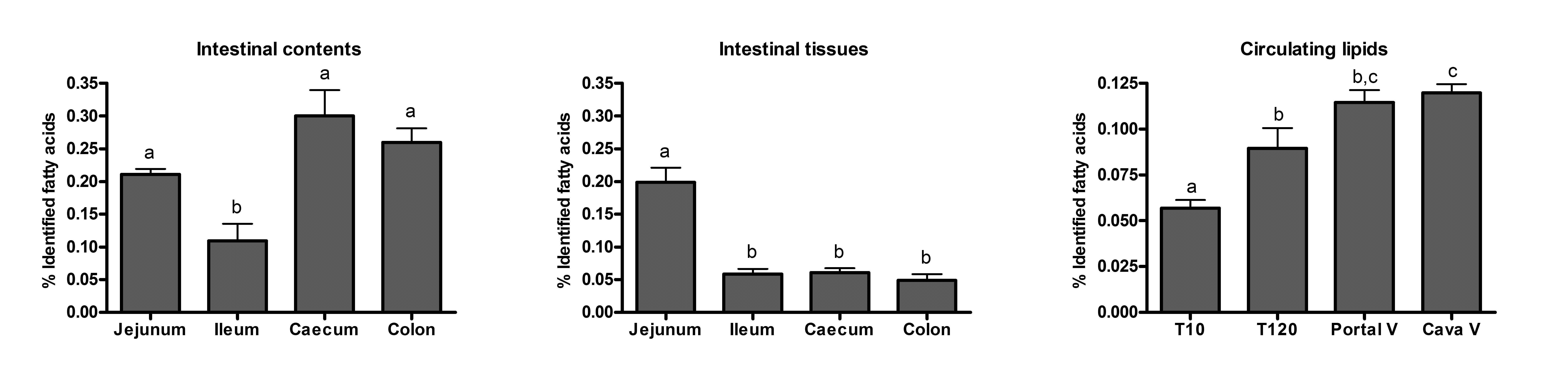

Supplement: Figure S1 — Sum of trans -9, cis -12-18:2 and trans -11, cis -15-18:2 in intestinal contents, intestinal tissues and circulating lipids of the sunflower oil force-fed mice. The results are expressed as a percentage of identified fatty acids. Data are mean ± SEM. Statistical significance of differences between intestinal contents, intestinal tissues and blood samples times were assessed by one-way ANOVA followed by Bonferroni's post-hoc multiple comparison test. Values with unlike superscript letters are significantly different (p<0.05). (TIF) [file pone.0087560.s001.tif]

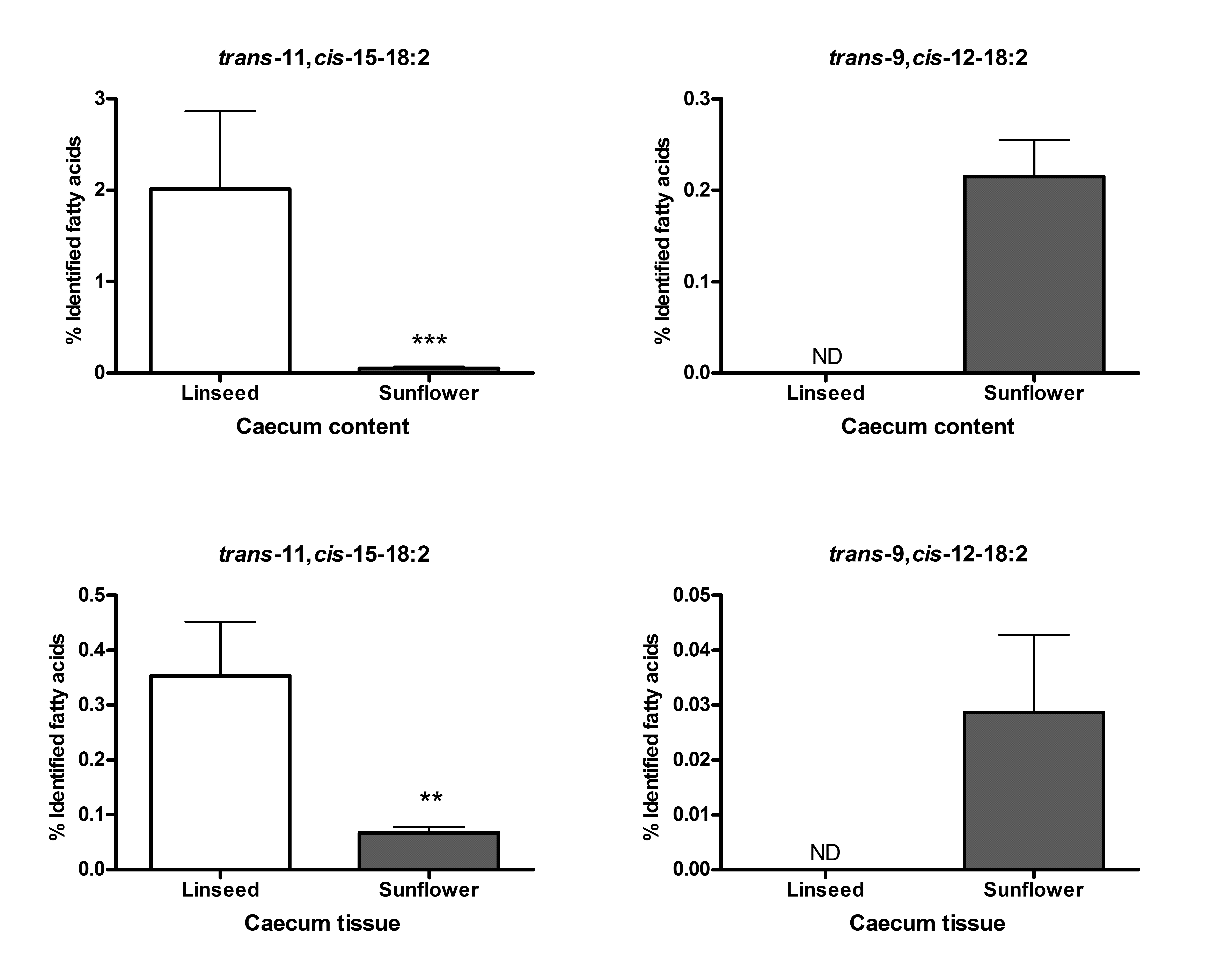

Supplement: Figure S2 — trans -9, cis -12-18:2 and trans -11, cis -15-18:2 in caecum content and caecum tissue analyzed by the GC method combining two temperature program. The results are expressed as a percentage of identified fatty acids. Data are mean ± SEM. Statistical significance of difference between oil force-feedings was assessed by Student t-test (** p<0.01; *** p<0.001). (TIF) [file pone.0087560.s002.tif]
